# Attracting, equipping and retaining young medical doctors in HIV vaccine science in South Africa

**DOI:** 10.4102/sajhivmed.v16i1.364

**Published:** 2015-11-19

**Authors:** Danna Flood, Melissa Wallace, Kimberly Bloch, James Kublin, Linda-Gail Bekker

**Affiliations:** 1 HIV Vaccine Trials Network, Fred Hutchinson Cancer Research Center, United States; 2 The Desmond Tutu HIV Centre, University of Cape Town, South Africa

## Abstract

**Background:**

HIV remains a significant health problem in South Africa (SA). The development of a preventive vaccine offers promise as a means of addressing the epidemic, yet development of the human resource capacity to facilitate such research in SA is not being sustained. The HIV Vaccine Trials Network (HVTN) has responded by establishing South African/HVTN AIDS Early Stage Investigator Programme (SHAPe), a programme to identify, train and retain clinician scientists in HIV vaccine research in SA.

**Objectives:**

The present study sought to identify factors influencing the attraction and retention of South African medical doctors in HIV vaccine research; to understand the support needed to ensure their success; and to inform further development of clinician research programmes, including SHAPe.

**Methods:**

Individual interviews and focus groups were held and audio-recorded with 18 senior and junior research investigators, and medical doctors not involved in research. Recordings were transcribed, and data were coded and analysed.

**Results:**

Findings highlighted the need for: (1) medical training programmes to include a greater focus on fostering interest and developing research skills, (2) a more clearly defined career pathway for individuals interested in clinical research, (3) an increase in programmes that coordinate and fund research, training and mentorship opportunities and (4) access to academic resources such as courses and libraries. Unstable funding sources and inadequate local funding support were identified as barriers to promoting HIV research careers.

**Conclusion:**

Expanding programmes that provide young investigators with funded research opportunities, mentoring, targeted training and professional development may help to build and sustain SA's next generation of HIV vaccine and prevention scientists.

## Introduction

Africa (SA) has one of the highest HIV prevalence rates globally.^1^ Despite promising new developments in non-vaccine prevention modalities, a preventive vaccine against HIV offers the best hope to end the epidemic.^2^

Several large-scale HIV vaccine, and other prevention, trials will begin in SA in 2016.^3, 4^ These trials will utilise the strengths of South African investigative teams, requiring a wide breadth of research capacity. Whilst senior-level South African HIV vaccine researchers have developed enormous expertise, the number of junior clinician investigators entering the field has lagged. Since the early 1990s, SA has seen a decrease in the number of clinical researchers and an ageing of publishing scientists.^5, 6^ A concerted effort is required to increase the number of young medical doctors entering the HIV prevention and vaccine field to ensure the country's ongoing contribution to this critical effort.

In 2010, responding to the challenge, the HIV Vaccine Trials Network (HVTN), with support from the National Institute of Allergy and Infectious Diseases (NIAID) and the Fogarty International Center (FIC), established the South African/HVTN AIDS Early Stage Investigator Programme (SHAPe), a peer-reviewed medical doctors/PhD programme that recruits and supports young clinician investigators as they become independent investigators. Components of the programme, coordinated by the Desmond Tutu HIV Centre at the University of Cape Town, include 3-year salaried appointments at HVTN clinical trial sites; financial support for a mentored research project and research-related costs; targeted training and tuition for a concurrent PhD programme; travel to HVTN meetings and international HIV conferences; and facilitated integration into the HVTN scientific community via participation on scientific and governance committees.

To inform the SHAPe programme and future efforts, formative research was conducted to: (1) understand facilitators and barriers to attracting, equipping and retaining young South African medical doctors in careers in HIV vaccine research, (2) develop recommendations to address identified challenges and (3) inform the design of clinician investigator development programmes, including SHAPe.

## Methods

### Sampling

During 2011, participants were recruited from clinical research sites and via referrals throughout SA. Target participants comprised a small group of specialised individuals and therefore purposive sampling with specific criteria was used. These groups were defined as: (1) senior investigators (SI) – those who led research teams and held the role of ‘principal investigator’ (PI) for at least 5 years, (2) junior investigators (JI) – those who had medical degrees and worked at clinical research sites for 10 years or less without assuming the role of PI and (3) young medical doctors (MD) – those who had received medical degrees no more than 12 years ago and expressed interest in research but had not pursued research careers.

### Data collection

We used a qualitative approach and conducted semi-structured interviews and focus groups. K.B. underwent comprehensive training and utilised interview and focus group schedules to guide the sessions. Fifteen one-on-one, semi-structured, confidential telephone interviews were conducted, lasting approximately 1 hour and included five JIs, five SIs, and five MDs. Data collected during the interviews informed semi-structured questions used for three face-to-face focus groups, lasting approximately 1.5 hours and included four SIs, three JIs and four MDs in separate groups. All interviews and focus groups were conducted in English, recorded using a hand-held audio recorder, and transcribed in English. Demographics questionnaires were completed by those who participated in focus groups.

### Data analysis

Three investigators independently coded five interview transcripts and then met as a team to review codes, resolve differences in coding by consensus, and create a codebook. The codebook was used to analyse subsequent interview and focus group transcripts, and generate new codes. Subsequent transcripts were assigned to three of the authors who independently cleaned and coded them using the codebook. The three investigators reconvened again to resolve differences in coding by consensus, analyse the transcripts, discuss patterns in the data and organise identifiable themes in ATLAS.ti.6.2.25 (GmbH Berlin). Data and investigator triangulation were utilised to enhance validity of the findings.^7^

## Ethical considerations

All participants were over 18 years old and provided written informed consent. The study was approved by the University of Cape Town's Human Research Ethics Committee (IR File #128/2011) and the Fred Hutchison Cancer Research Center Institutional Review Board (IR File #7448).

## Results

Participants (*N* = 18) were SIs (*n* = 5), JIs (*n* = 6), and MDs (*n* = 7). Sociodemographic characteristics of study participants are shown in Table 1.

The following themes were identified for each research objective and were endorsed by participants from all groups (JI, SI, MD) unless otherwise stated.

**TABLE 1:** Sociodemographic characteristics of study participants.

Sociodemographic characteristics	n	%
Gender†		
Male	4	22
Female	14	78
Race or cultural group		
Black	6	33
Mixed race	1	5.5
Asian	2	11
White	3	17
African or Euro-African	5	28
Non-response	1	5.5
Employment category		
Medical doctor	7	39
Junior investigator	6	33
Senior investigator	5	28
Age, mean‡	42	8.52
SD	30–53	-
Total participants	18	100

† , *N* = 18; ‡, *n* = 17.

### Attracting young doctors to research

#### Exposure to research early in medical training:

Most participants believed there is little exposure to research in medical training and a majority of young doctors are not aware that research is a viable career option:

‘[*Clinical research*] is an unconsidered job because … it's not explored in medical school.’ (MD)

Many participants indicated that even brief exposure to research (e.g. an investigator presenting their research to students and professors) can spark interest in a research career. There was wide agreement that incorporating research methodology courses and hands-on research with medical training early in the curriculum would help doctors to better understand research and fuel interest in this career option:

‘… integrate clinical researchers back into [*university*] departments. … people … could see … what fun research is and how research changes people's lives and become excited about doing this…’ (SI)

Suggestions for supporting these activities included providing students with small mentored research projects, offering stipends to support projects, and combining a medical degree with a master's or PhD degree.

Several participants indicated that better integration of researchers into university academic departments would provide them with greater access to funding support, training and academic resources, whilst providing medical schools and other departments with faculty to teach research methodology, and opportunities for students to gain research experience:

‘Clinical researchers are incredibly isolated in the university; even though they are driving the publications of the university and … the research agenda … [*they*] are isolated from [*their*] peers.’ (SI)

#### Career development pathway:

Most participants, particularly MDs, highlighted a lack of clarity around the career development pathway for clinical researchers as a deterrent to pursuing a career in this field:

‘… there isn’t really a … [*formal*] progression… There's no hierarchy. Either, you’re … the PI or you’re not the PI …’ (JI)

This is in contrast to the well-defined and regimented career pathway for clinical medicine:

‘[*In clinical medicine*]… determining your path is much more strict and clearly defined… [*clinical research*] has a lot of uncertainties…’ (MD)

#### Comparable employment packages and job security:

Nearly all participants agreed that, to attract and retain top research doctors, employment packages in clinical practice and research need to be comparable. Perceptions of researcher v. non-researcher salaries varied. To the best of our knowledge, there are no definitive data comparing salaries of medical doctors in clinical practice v. research. All SIs agreed that salaries have been historically lower for researchers, but recent efforts have been made to align salaries more closely; increasing awareness of this trend could help to attract doctors to research:

‘… making comparable salaries [*to doctors in clinical practice is*] important … We are really struggling to find doctors [*to bring*] into research…’ (SI)

#### Increase awareness of the HIV prevention field:

Many investigators reported that the subject of HIV prevention sparked their interest in research, citing the urgency and magnitude of the HIV epidemic and their desire and ability to make a difference in people's lives. Many participants suggested that increasing awareness amongst the medical community about developments in HIV prevention modalities, particularly vaccines, could stimulate interest in the field. Suggestions included presentations at medical conferences and in media frequented by clinicians:

‘… show [*doctors*] what the future holds for HIV/AIDS. To be able to see … the result, the impact in the community … it can be very satisfying for a career.’ (JI)

### Equipping and retaining doctors for careers in research

There was considerable overlap in themes identified as important for equipping and retaining doctors; therefore they are combined here.

#### Skill building, infrastructure and academic resources:

To effectively equip JIs, SIs noted that they need specialised training. Most participants highlighted the importance of acquiring research knowledge and skills through academic courses in ‘… epidemiology, statistics, immunology… ethics … [*and*] research methods…’ (SI). Some suggested courses in ‘laboratory methods.’ Most SIs recommended intensive training in ‘… manuscript and proposal writing …’ and ‘[*giving*] presentations’. Participants also mentioned the importance of collecting data in the field and working in research units.

Nearly all participants agreed that, to train and retain new investigators, the investigators need access to academic resources such as journal subscriptions, educational opportunities, research symposia and networking to foster ‘collaboration … [*with a*] wider scope of people …’ (SI).

#### Mentorship:

Structured mentoring was raised by most participants as a significant facilitator to equipping, retaining and supporting career progression for new investigators.

Nearly all participants proposed that fostering an environment with structure, clear objectives, dedicated time, and positive personal relationships is essential for a successful mentor-mentee experience:

‘[*we need a*] formal structure … the mentor and the mentee *[should*] draw up a contract that spells out … purpose… goals and targets … time frames … to guide this relationship.’ (JI)

Despite the acknowledged importance of mentorship, most participants identified that lack of time hinders the mentoring process. All SIs emphasised that sufficient time for mentoring requires specific funding allocated for mentorship activities, but acknowledged the difficulty of earmarking research funding for mentorship.

Many investigators noted that they themselves had not been mentored and, as a result, some SIs conceded that they did not feel capacitated for this role. Several participants suggested the development of a mentorship training programme for all investigator levels to address this challenge:

‘… we need support. … mentors need coaching about how to be a good mentor because … [*we’ve*] never had one.’ (SI)

Participants noted the importance of the JI and SI relationship in ensuring career progression and enhancing retention in the field. JIs mentioned the critical role that SIs play in providing linkage to resources, people and networks which can assist them in furthering their career. Most SIs and JIs recognised that if an SI was unwilling or unable to facilitate JI development and ‘make space at the top’, it could jeopardise career progression:

‘… if that chief person [*the SI*] … has no spirit of “Look, I’m on top, now I can pull you up to come join me.” If that doesn’t exist, it's a serious barrier.’ (JI)

#### Adequate funding for research:

All SIs and JIs expressed concerns about the inherently unstable nature of grant funding and thought it deterred doctors from entering, progressing in, and retaining research careers because, in contrast, clinical practice salaries are thought to be ‘guaranteed’. In addition, the limited availability of consistent and sustained government and university research funding in SA was a frequent theme:

‘If you look at research, in general it's something that's run by donors, and if you go into that kind of career … you are not sure whether that job is secure…’ (JI)

Because much research funding is sourced internationally and tends to be on a project or grant basis, the ability for long-term planning is affected. Financial concerns are perceived to hamper career progression, because the need to secure funding is constantly prioritised above the ‘science’:

‘… instead of spending my time thinking what's the next interesting research question, I’m thinking how the hell do I keep this funded? Sure, that dilutes efforts quite a lot.’ (SI)

Some participants suggested seeking alternative funding sources, particularly from the South African government, to establish a more stable funding base to supplement grants:

‘If the government is proactive in leading research … the private sector will also move in and pour in funds when they see there is commitment from the government.’ (JI)

### Clinician investigator development programmes

Most participants believed that it was critical for the clinical research field to create more clinical investigator development programmes that provide structured mentored research, training, and professional development opportunities:

‘[*Programmes like SHAPe offer*] something that is necessary. … people have to be given this opportunity, otherwise I don’t see them paying their way through this kind of programme…’ (JI)

Several participants emphasised that development programmes must provide the opportunity for young investigators to experience the full range of activities, and hone the practical skills needed to be a successful investigator:

‘You’re gonna need to give people the experience of doing all of it … if they’re going to become the next generation PIs they do actually need to have that exposure and be able to do it.’ (JI)

‘… things like regulatory [agency research review requirements], to understand how that process works … the practical experience of how to go about that.’ (SI)

## The South African/HVTN AIDS Early Stage Investigator Programme

Participants were asked to reflect on the design of the SHAPe programme and comment on the strengths and weaknesses of this model, and provide input on ways to improve the programme.

Most participants were enthusiastic about the SHAPe programme and agreed that it served an important role in providing an opportunity not previously available: to recruit and train young doctors in research. Participants identified SHAPe's strengths as providing a mentored research project and full-time salaries, incorporating a PhD degree programme, offering experience as a clinical trial doctor, and opportunities for travel to research meetings and conferences. Lack of awareness of the programme by the medical community was often identified as the most significant weakness:

‘… I’m actually thinking about [*applying to SHAPe*] … Nobody approached me before personally and said … this is what it entails … are you interested? Personal contact is the best [*way to recruit*].’ (MD)

Participants suggested several ways to improve SHAPe. Most advocated greater involvement from SIs and JIs in publicising the programme and presenting research findings at medical schools and hospitals to increase interest in HIV vaccine research.

Most participants agreed that the best recruitment approach is multi-pronged and includes ‘personal outreach’, ’national publicity’ and advertising through the ‘provincial Departments of Health’, ‘newspapers’, ‘major medical journals’ and ‘medical association websites’. A few participants also advocated use of social media such as Facebook (SI), direct SMS (MD) and email (MD).

Some participants suggested expanding the programme to support independent research for mid-career HIV vaccine investigators. A few suggested increasing the number and location of sites where scholars can work.

## Discussion

The present study identified factors influencing the attraction and retention of South African medical doctors into HIV prevention research; increasing the understanding of the resources and support needed to ensure their success; and eliciting suggestions to inform design of clinical research development programmes, such as SHAPe.

Of note is that findings were largely applicable to HIV prevention research generally and clinical research more broadly, increasing their value and impact in informing recommendations and future practice. Overwhelmingly, there was recognition from all groups that research was not adequately represented in the medical training curriculum and that this limited exposure discouraged interest in, understanding of, and entrance into this field. The overall shortage of doctors in primary healthcare is critical in SA^8,9, 10^ and requires urgent redress.^11^ As a result, medical education has renewed its focus on primary healthcare.^12,13^ Whilst necessary, this focus on primary healthcare has had a detrimental effect on the development of clinical research capacity and solid grounding in research skills^12^; and although medical schools may include training on evidence-based medicine, it is not prioritised as a critical part of the curriculum.

Our study's findings highlight the need for a more clearly defined career pathway for MDs entering research, which includes research skill development, senior researcher mentorship, academic resource access, and networking opportunities. These findings mirror those of the Academy of Science of South Africa (ASSAF) report, which reviewed the overall state of clinical research in SA and indicated the inadequacies of these resources for clinical research.^12^

A major barrier to the attraction, retention and career progression of doctors in research is the perceived lack of secured and ongoing funding. With limited financial support from the South African government and academic institutions, the large amounts of money required to conduct HIV prevention trials are sourced outside SA. This project-based funding is competitive, subject to exchange rate fluctuations, and often short-term and unstable.

Based on these findings, several recommendations can be made.

Exposure to research:

- Enhance academic research modules, and develop more programmes where students at all levels can gain hands-on experience to spark interest in clinical research, including HIV vaccine research.
- Expand the number of programmes offering a combined MBBCh and PhD degree.
- Foster an awareness of research, particularly HIV prevention research and its impact, amongst both medical professionals and the broader community.

Training and mentorship:

- Develop coordinated training programmes for HIV clinical researchers at all levels, as well as a structured mentorship programme supporting researchers vertically and horizontally. Provide training on effective mentoring for JIs and SIs.
- Develop a more objective, standardised and clearly defined career development pathway for JIs with clear expectations, milestones and incentives (e.g. promotions, awards) and promote it widely.

Funding:

- Increase local/national funding (e.g. government, industry, corporate, donor).
- Address funding allocation to support projects, the researcher and designated time for mentorship activities.
- Ensure that salaries are comparable across research and clinical practice positions, and publicise this widely.

Clinical investigator development programmes:

- Expand programmes such as SHAPe that provide comprehensive support. Promote via personal outreach in medical schools, registrar programmes, hospitals and conferences. Advertise in journals, social media, medical bulletins, SMS and email from trusted sources.
- Create advancement opportunities for JIs. For example, create new PI roles at research sites, faculty positions and opportunities for independent research.
- Incorporate JI development programmes in networks, universities and other collaborative research environments to leverage research and academic infrastructures.

### Study limitations

There are several limitations to the present study. The sample size was small, comprising 18 participants, most of whom took part in both one-on-one interviews and focus groups. Whilst this is considered an adequate sample size for qualitative research,^14^ it possibly limits the generalisability of the present results. However, notably two of the three groups included in this study (SIs and JIs) are, by definition, small in size. HIV vaccine research is a specialised field and it is partly because of the small number of doctors entering this field that the present research was conducted. Consequently, particularly for SIs, our sample included a significant proportion of the total SI population involved in HIV vaccine research in SA.

In addition, given the small size of this research community in SA, it is possible that participants did not feel comfortable speaking freely and honestly, out of concern that they would be recognised, despite removal of identifiers. This risk might have been an issue particularly during focus groups, as many investigators are colleagues and collaborators. Nevertheless, all participants expressed an interest in and recognition of the importance of the topic, and were aware of the value of the research process, and it is hoped that this encouraged openness and honesty throughout.

Finally, factors identified by participants as significant in attracting, equipping and retaining doctors in HIV prevention science research were largely representative of their own needs rather than a broader impression of what might be required overall. Whilst inevitable, this may be viewed as a limitation.
